# Invasive alien species and disease risk: An open challenge in public and animal health

**DOI:** 10.1371/journal.ppat.1008922

**Published:** 2020-10-22

**Authors:** Eleonora Chinchio, Matteo Crotta, Claudia Romeo, Julian A. Drewe, Javier Guitian, Nicola Ferrari

**Affiliations:** 1 Department of Veterinary Medicine, Università degli Studi di Milano, Milan, Italy; 2 Veterinary Epidemiology, Economics and Public Health Group, Royal Veterinary College, Hawkshead Lane, North Mymms, Hatfield, Hertfordshire, United Kingdom; University of Wisconsin Medical School, UNITED STATES

## Why we should care about invasive alien species from a health perspective

The anthropogenic movement of pathogens into new geographic locations or host species, so-called “pathogen pollution” [1], is one of the main threats to human and animal health in a globalized world.

Since the majority of zoonotic emerging diseases originate from wildlife [2], as recent outbreaks like Severe Acute Respiratory Syndrome Coronavirus 2 (SARS-CoV-2), Nipah, or Chikungunya point out, particular attention should be paid to wild animals’ translocations, which represent a potential driver of change in pathogen ecology and distribution [1].

Invasive alien species (IAS) are species of animals, plants, fungi, or microorganisms translocated by humans into environments outside their natural range, in which they establish and spread, negatively affecting the dynamics of local ecosystems. They are characterized by rapid reproduction and growth, high dispersal ability, and high adaptability to new conditions, thus often outcompeting native organisms in their introduced range [3], and have been recognized as one of the main causes for biodiversity loss globally [4]. Some well-known examples of IAS include the south-American coypu *Myocastor coypus*, invasive in North America, Europe, and Asia, where it causes both environmental and economic impacts consuming aquatic vegetation and undermining riverbanks [5], and the eastern-Asiatic brown marmorated stink bug *Halyomorpha halys*, a successful global invader causing severe economic damages to agricultural crops [6].

Besides affecting biodiversity conservation and economy, IAS, as translocated species, may promote pathogen pollution in the invaded area leading to the emergence of diseases [1,7–9]. It would thus be fair to expect animal IAS to be the focus of intense study by epidemiologists with regard to their disease risk toward native animals (both wild and domestic) and humans, as most of them thrive in anthropogenic environments, potentially increasing the risk for zoonotic pathogen emergence [9].

Within the field of invasion ecology, there has been a wide interest in exploring the relationships between invasions and infections during the last decades. Researchers focused in particular in understanding how parasites (or the lack of them) may facilitate or hamper the invasion process [10–13], how co-introduced parasites may themselves succeed in becoming invasive [13–15], and explored the effects that IAS may have on native parasites dynamics [13,14,16–18]. However, outside the invasion ecology field, IAS have yet to gain attention among people working in the fields of animal and public health, and the concepts explored in the ecological context cannot always find application in the development of health initiatives aimed at protecting public and animal health. For example, empirical research on IAS pathogens, which would be needed to assess the risk of infectious disease emergence, is skewed toward a few species (e.g., vector species like the tiger mosquito *Aedes albopictus*) or toward selected pathogens known to harm biodiversity conservation, while a global vision of IAS-associated health threats is still not available [9,19–21].

In this context, it is urgent to raise awareness in people working in the fields of animal and public health of the need to consider IAS as a health threat. To this aim, we provide here an overview of how animal IAS may affect local disease dynamics both directly and indirectly, i.e., acting as pathogen hosts or disrupting the recipient ecosystem structure, through real-case examples from the ecological literature, and, in the last paragraph, we propose future initiatives aimed at improving our capacity for targeted actions toward the IAS most likely to threaten human and animal health, calling for an increased involvement of people working in the fields of animal and public health in a new invasion epidemiology field.

## IAS as sources of new pathogens

IAS may host pathogens that are absent in the area of release and cause their establishment and subsequent spillover to local species, possibly resulting in an increase of disease risk for humans, domestic animals, and native wildlife.

**Fig 1:**
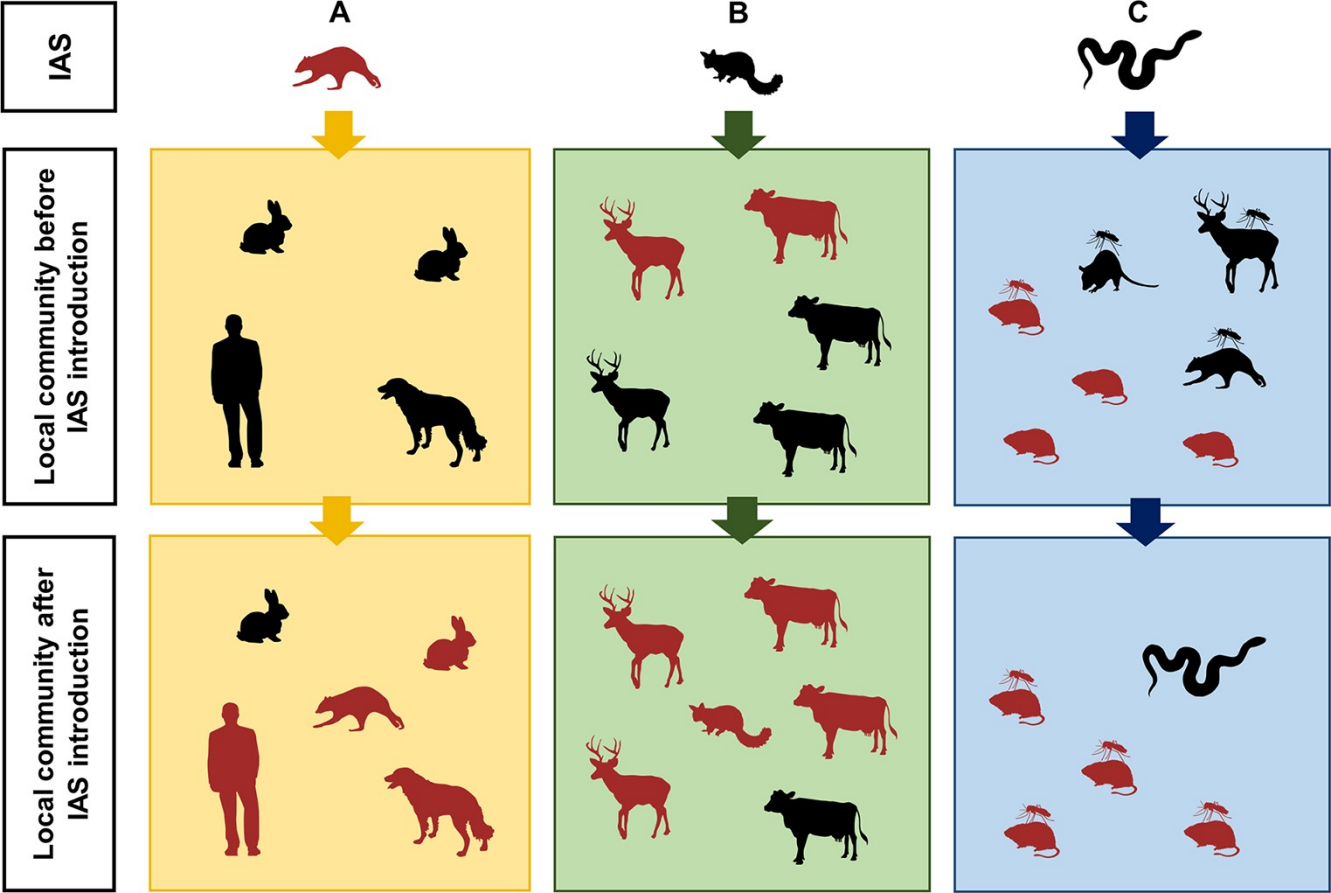
Mechanisms through which IAS may increase disease risk: Real-case examples. Dark red silhouettes represent infected hosts, and black silhouettes represent uninfected hosts. (A) IAS as sources of new pathogens: the north-American raccoon *Procyon lotor* introduced the nematode *Baylisascaris procyonis* into central European countries. Raccoons are the definitive host for *B*. *procyonis*, and they contaminate the environment by shedding parasite eggs through feces. Small mammals and birds may serve as paratenic hosts, while domestic dogs may rarely act as alternative definitive hosts. Humans, which acquire the infection as accidental hosts, can develop severe symptoms, caused by larval migration to tissues. (B) IAS as amplifiers of local pathogens: the invasive Australian possums *Trichosurus vulpecula* became the main reservoir host for bovine tuberculosis in New Zealand. Despite *Mycobacterium bovis* being introduced to New Zealand via cattle in the 1800s and possums in the 1850s, the disease was detected in possum populations only in the 1970s, in locations occupied by wild deer, when decapitation of deer was a common hunting practice. Intensive possum control actions, which cost to the country about $NZ50 million per year, have so far produced huge reductions in the number of infected cows and deer, but New Zealand is not yet free from the disease. (C) Indirect mechanisms by which IAS can disrupt local infection dynamics: in Florida, invasive pythons *Python bivittatus* reduced the abundance of several large and medium-sized mammals, indirectly causing the redirection of the mosquito vectors for the zoonotic Everglades virus from low-competent hosts, like deer, raccoons, and opossums, to the main reservoir host, the hispid cotton rat *Sigmodon hispidus*. Further research is needed to assess if the increased abundance of infectious vectors corresponds to an increase of disease risk for local human populations. IAS, invasive alien species.

The north-American raccoon *Procyon lotor*, for example, introduced to Central Europe *Baylisascaris procyonis* [22], a nematode causing *larva migrans* syndromes potentially inducing severe central nervous system disease in humans (Fig 1A). Introduction to Europe of north-American crayfishes *Procambarus clarkii* infected with the fungus *Aphanomyces astaci* caused huge economic losses to fisheries, being the pathogen lethal to native crayfishes [23]. Similarly, squirrelpox virus, introduced to the United Kingdom along with the American eastern gray squirrel *Sciurus carolinensis*, is significantly contributing to the increased mortality of native red squirrels *Sciurus vulgaris* [24].

However, while pathogen co-introductions occur over a wide range of parasite and host taxa [14], some pathogens are lost during the invasion process [25]: for example, there is no evidence for Poxvirus in Italian gray squirrel populations [26]. Pathogen loss may be due to the absence of the pathogen in the individuals of the founding populations or to its inability to survive to translocation or establish in the area of release. The outcome depends on several factors related to the IAS (e.g., founding population origin), the pathogens (e.g., host specificity), and the area where the species is released (e.g., environmental conditions, presence, and density of local hosts) [15]. As shown by a study on ectoparasites of introduced birds, factors related to transmission efficiency, such as the number of host introduced and host longevity, are likely to play a major role [15].

## IAS as amplifiers of local pathogens

An increase of local disease risk may also occur if the introduced IAS is susceptible to, and able to transmit, local pathogens. Pathogens acquired by IAS may be amplified and possibly spill back to humans and local species [27].

A case in point is the Australian brushtail possum, *Trichosurus vulpecula*, in New Zealand (Fig 1B). Invasive possums probably became infected with *Mycobacterium bovis*, the causal agent of tuberculosis in cattle, from wild deer, after the beginning of commercial deer hunting in 1960. Currently, they are the most important maintenance host for bovine tuberculosis, supporting higher transmission rates compared to local species and, being sympatric with cattle, providing interface for transmission between livestock and forest residents [28]. Another case is represented by invasive raccoon dogs *Nyctereutes procyonoides*, which may amplify rabies circulation in Eastern Europe or cause its reemergence in currently rabies-free countries [29].

IAS competence for pathogen transmission plays a major role in defining the outcome of pathogen acquisition, and, as the possum–tuberculosis case exemplifies, it is the result of both IAS–pathogen interaction (e.g., IAS susceptibility, period of communicability, and pathogen excretion rate) and IAS behavioral patterns (e.g., habitat, home range extension, and intra- and interspecific contact rates).

Based on IAS competence, the acquisition of a local pathogen may even lead to the reduction of disease risk (the so-called dilution effect [30]) or to no consequences at all. For example, in Ireland, the invasive bank vole *Myodes glareolus* has been found to divert fleas from the native wood mice *Apodemus sylvaticus*, which is a more competent host for *Bartonella* spp. [31]. However, the identification of the contexts in which a dilution effect may occur is still highly debated in ecology, as it strongly depends on local host species diversity and on the interactions occurring between the species involved in the transmission cycle [30].

## Indirect mechanisms by which IAS can disrupt local infection dynamics

Introduced species may disrupt local infection dynamics also indirectly, i.e., nonacting as pathogen hosts but through competitive and trophic interactions with native species or modification of local habitats, thus altering the abundance and/or contact rates among local host species, parasite infective stages, or vectors.

In southern Florida, the invasive python *Python bivittatus* caused the decrease of several mammal species, inducing the local mosquito vector of zoonotic Everglades virus to feed almost exclusively on the virus’ main reservoir host, the hispid cotton rat *Sigmodon hispidus*, potentially leading to an increase in pathogen circulation (Fig 1C) [32]. An example of habitat alteration is given by the activity of invasive feral pigs *Sus scrofa* on the island of Hawaii: they create wallows and cavities in tree fern trunks improving habitat suitability for mosquito vectors for avian malaria *Plasmodium relictum* [33], one of the main threats to native Hawaiian forest birds’ conservation.

Again, IAS indirect effects on local infection dynamics are highly context dependent, and mechanisms presented so far may act in concert, producing unpredictable outcomes. In Scotland and Northern England, for example, the invasive gray squirrel has been found to harbor several local strains of *Borrelia burgdorferi* [34]. However, in those areas, gray squirrels are also causing the decline of another competent host for *B*. *burgdorferi*, the red squirrel, and the effect of these concurring mechanisms on human Lyme disease risk remains unknown [34].

## A call for action: From invasion biology to invasion epidemiology

During the last centuries, more than 16,000 IAS introduction events have been recorded worldwide, and this number still presents an increasing trend [35]. In such context, the identification of those species deserving priority attention, based on their actual and potential impacts, is essential to support decision-making [36]. Several tools to inform preventive and management actions on animal IAS, including horizon scanning protocols, risk assessments, and impact assessments, have been developed in the last years (see [37] for a recent review), but the majority of them focus on environmental impacts, not specifically considering disease emergence risks in humans and local animal populations [38,39]. Some authors have called for a greater attention on the potential health risks posed by biological invasions [7–9,21], highlighting the need for a better integration between biological and health sciences, surveillance actions, and coordinated policies. We support their appeal, arguing that an increased awareness of people working in the fields of animal and public health on the risks concerning biological invasions and their consequent involvement in the invasion biology field is the first step toward a complementary invasion epidemiology field. Such field would be integrated with invasion ecology, but more specifically aimed at the prevention of the emergence of diseases in human and animal populations consequent to IAS introduction and establishment. To this aim, we propose some initiatives that should be addressed by future research work.

A first major constraint in addressing the issue of disease emergence connected to IAS is given by the lack of comprehensive data on pathogens affecting IAS. In this sense, we recommend the gathering in ad hoc databases of all the available information on IAS pathogens affecting human and animal health, including their geographical distribution and prevalence in IAS populations, in both native and introduced ranges.

It would also be advisable to improve our understanding of the key epidemiological events and factors driving the emergence of infectious diseases following IAS establishment, for example, through ex-post analyses on the already established IAS. In particular, as the emergence process of a disease is composed of several stages (introduction in a new area/host population, establishment, and spread) [14,40,41], the key factors involved in the process and related to IAS biology, pathogenic features, and the biotic and abiotic components of the area of release should be identified for each of these stages.

We also suggest urgently directing research efforts at developing transparent and flexible tools able to prioritize IAS based on the risk of transmitting pathogens with the potential to impact the health of humans, production animals, and native wildlife. Such tools could be based on the framework of the World Organisation for Animal Health (OIE)/International Union for Conservation of Nature (IUCN) for wildlife disease risk analysis and readapted to account for the main mechanisms through which alien species may affect local health, in particular the introduction of new pathogens and the acquisition and spread of local ones. The lack of data on IAS pathogens is certainly an obstacle in underpinning in-depth risk assessments [22], in particular, quantitative ones. However, a simple and transparent qualitative disease risk assessment procedure would enable the prioritization of empirical research needed to cover these knowledge gaps, while at the same time guiding local health administrators in the allocation of resources for management and preventive actions toward IAS. The issue related to irregular data availability could be partially overcome, as a first step, by eliciting opinions from experts.

Finally, awareness and action will be influenced by, and need to consider, the wider public perspective, not just researchers and institutions. Initiatives aimed at sensitizing citizens about the health threats of IAS will be needed to promote responsible behaviors when crossing borders and to improve the general public attitude toward IAS control and eradication programs.

All the suggested initiatives, to be successful, necessitate a stronger connection between ecologists, biologists, and other people working in the fields of animal and public health and beyond. Only through wider collaboration and dialogue will the potential health impacts of biological invasions be fully appreciated and, perhaps, ameliorated.
